# Exploring the Effectiveness and Durability of Trans-Kingdom Silencing of Fungal Genes in the Vascular Pathogen *Verticillium dahliae*

**DOI:** 10.3390/ijms23052742

**Published:** 2022-03-01

**Authors:** Tao Zhang, Jian-Hua Zhao, Yuan-Yuan Fang, Hui-Shan Guo, Yun Jin

**Affiliations:** State Key Laboratory of Plant Genomics, Institute of Microbiology, Chinese Academy of Sciences, CAS Center for Excellence in Biotic Interactions, University of the Chinese Academy of Sciences, Beijing 100049, China; tzhang@im.ac.cn (T.Z.); zhao_jian_hua@hotmail.com (J.-H.Z.); fangyy@im.ac.cn (Y.-Y.F.)

**Keywords:** host-induced gene silencing, trans-kingdom RNA interference, *Verticillium dahliae*

## Abstract

Host-induced gene silencing (HIGS) based on trans-kingdom RNA interference (RNAi) has been successfully exploited to engineer host resistance to pests and pathogens, including fungi and oomycetes. However, revealing the mechanisms underlying trans-kingdom RNAi between hosts and pathogens lags behind applications. The effectiveness and durability of trans-kingdom silencing of pathogenic genes are uncharacterized. In this study, using our transgenic *35S-VdH1i* cotton plants in which dsVdH1-derived small RNAs (siVdH1) accumulated, small RNA sequencing analysis revealed that siVdH1s exclusively occur within the double-stranded (ds)VdH1 region, and no transitive siRNAs were produced beyond this region in recovered hyphae of *Verticillium dahliae* (*V. dahliae*). Accordingly, we found that *VdH1* silencing was reduced over time in recovered hyphae cultured in vitro, inferring that once the fungus got rid of the *35S-VdH1i* cotton plants would gradually regain their pathogenicity. To explore whether continually exporting dsRNAs/siRNAs from transgenic plants into recipient fungal cells guaranteed the effectiveness and stability of HIGS, we created GFP/RFP double-labeled *V. dahliae* and transgenic *Arabidopsis* expressing dsGFP (*35S*-*GFPi* plants). Confocal images visually demonstrate the efficient silencing of *GFP* in *V. dahliae* that colonized host vascular tissues. Taken together, our results demonstrate that HIGS effectively triggers long-lasting trans-kingdom RNAi during plant vasculature *V. dahliae* interactions, despite no amplification or transitivity of RNAi being noted in this soil-borne fungal pathogen.

## 1. Introduction

RNA interference (RNAi, or RNA silencing) is universal in eukaryotes, including animals, plants, and fungi. Compared with RNAi in animals and plants, the mechanisms of fungal RNAi are diverse based on studies in the model fungus, *Neurospora crassa* [1,2,3]. The existence of RNAi in plant phytopathogenic fungi is largely unknown [4,5,6]. In a few studies on *V. dahliae* [7], *Botrytis cinerea* [8,9], *Fusarium* [10,11,12], *Magnaporthe oryzae* [13,14,15,16,17,18], *Puccinia* [19,20], and *Valsa mali* [21,22,23], small RNAs (sRNAs) and microRNA (miRNA)-like sRNAs have been sequenced, and RNA silencing-related genes have been cloned. However, the function and mechanism of the regulation process have not been completely determined. RNAi in fungi is significantly diversified, and the numbers of RNA silencing proteins differ considerably among fungal species [24]. This diversification may lead to reduced efficacy of RNAi in some fungal species, such as *Ustilago maydis*, in which the entire RNA silencing machinery appears to have been lost [25]. 

Phytopathogenic fungi cause huge yield losses to crops worldwide. In addition to fungicides, which represent current approaches to control phytopathogenic fungi, alternative approaches based on RNAi named HIGS or spray-induced gene silencing (SIGS) are designated as in vivo approaches and in vitro approaches, respectively [26,27,28,29,30,31]. dsRNA or hairpin RNA (hpRNA) are introduced into host plants or through exogenous application to produce siRNAs targeting the essential genes of phytopathogens. In contrast to the few studies on the RNA silencing pathways of fungi, it has been demonstrated that this trans-kingdom RNAi approach is efficient in defending against many phytopathogenic fungi, such as *Blumeria graminis* [32] *V. dahliae* [33,34], *B. cinerea* [35], and *M. oryzae* [36]. Nevertheless, there is still an exception that transient HIGS is inefficient in inducing silencing in *Zymoseptoria tritici*, which is unable to take up dsRNA; however, this species encodes the core components of RNA silencing proteins [37,38]. Hence, for better application in crop protection, the mechanism and efficiency of these trans-kingdom siRNAs in fungal cells need to be investigated. Plants send miRNAs to fungal pathogens to silence virulence genes, indicating that natural trans-kingdom RNAi exists in host–pathogen interactions [39,40]. Further studies revealed that plants send sRNAs in extracellular vesicles to fungal pathogens to silence virulence genes [41]. Recently, endocytosis appeared to facilitate the uptake of dsRNA, as demonstrated in *B. cinerea* [40] and *Sclerotinia sclerotiorum* [42]. 

For soil-borne vascular fungi, the uptake of in vitro synthesized dsRNA seems invalid or less efficient due to the instability of naked RNA in the soil. However, pretreatment of *Arabidopsis* roots with dsRNA potentially inhibits the infection of *V. dahliae* [43]. HIGS, which is laborious but more stable, is still an efficient and competitive approach. Therefore, understanding the mechanisms of HIGS, for instance, in planta-delivered dsRNA or siRNAs concerning the spreading and efficient location of siRNAs, is critical in applying this profound approach for controlling pathogenic fungi, especially soil-borne fungi. We previously created transgenic cotton *35S-VdH1i* expressing hairpin RNA specific to the hydrophobin (*VdH1*) gene of *V. dahliae,* a soil-borne vascular fungal pathogen [33]. These *35S-VdH1i* cotton plants exhibited highly effective resistance to *V. dahliae*. The degradation of *VdH1* mRNA and the accumulation of VdH1-derived siRNAs in hyphae recovered from infected transgenic cotton indicated plant-delivered siRNAs entered into and induced silencing in fungal cells [33]. Later, in tomato and *Arabidopsis*, HIGS was also demonstrated to be effective in defending against Verticillium wilt [34]. These results demonstrate the effectiveness of HIGS on *V. dahliae,* thereby conferring resistance to wilt disease in host plants. Three RNA-dependent RNA polymerases proteins (RDRs) are predicted in *V. dahliae* [7]. This prompted us to investigate whether these fungal RDRs would induce siRNA amplification of VdH1-derived siRNAs. In this study, through small RNA sequencing and Northern blot analysis, we found that fungal RDRs were not involved in the amplification of trans-kingdom RNAi signaling. By creating a visible silencing system, we further demonstrated that the highly effective trans-kingdom silencing of fungal targets took place inside the infected plants. All these results indicate that without amplification of RNAi, the efficacy of trans-kingdom silencing relies on persistent transporting dsRNA/siRNA in vascular tissues where *V. dahliae* colonized.

## 2. Results

### 2.1. Transitive Silencing of the Trans-Kingdom Does Not Occur in Recipient V. dahliae

In plants, sRNA-mediated cleaved mRNAs may recruit RDRs to generate more dsRNAs and produce secondary siRNAs from regions surrounding the primary silencing trigger sequence, a silencing phenomenon termed silencing transitivity [44,45]. We have recently identified three RDRs in *V. dahliae* [7]. To investigate whether three fungal RDRs were involved in trans-kingdom RNAi for secondary siRNAs production in recipient cells, we utilized *35S-VdH1i*-cotton line 3, which produces *VdH1*-derived siRNAs (siVdH1) [33] for infection with *V. dahliae* V592 and fungal recovery. The fungal hyphae recovered from wild-type and *35S-VdH1i* cotton plants were named Vd^WT−1st^ and Vd^VdH1i−1st^, respectively. Small RNAs isolated from Vd^WT−1st^ and Vd^VdH1i−1st^ colonies as well as *35S-VdH1i* transgenic cotton plants were sequenced. siRNAs mapped to *VdH1* were included in our analysis. 

**Figure 1:**
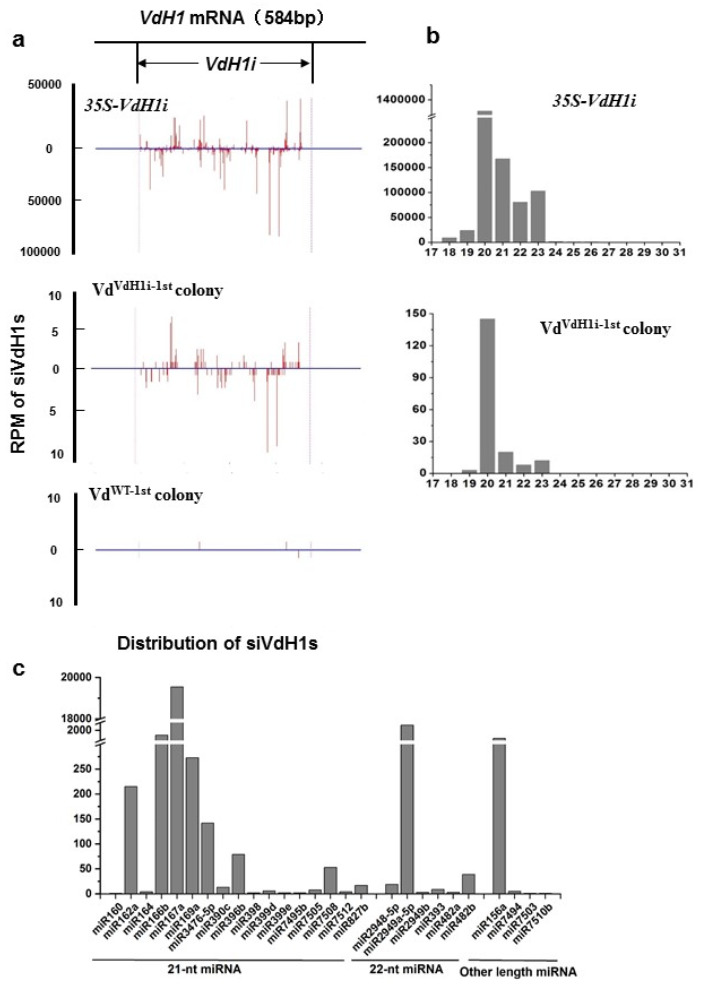
Small RNA analysis of *35S-VdH1i* transgenic cotton, Vd^VdH1i−1st^, and Vd^WT−1st^ colonies. (**a**) RPM (reads per million sequences) and distribution of siVdH1s obtained by deep sequencing. The 5′-terminal 380bp VdH1i region is indicated. The y-axes represent the RPM of siVdH1s in small RNA libraries of *35S-VdH1i* cotton, Vd^VdH1i−1st^ colonies, and Vd^WT−1st^ colonies related to positions along the *VdH1* gene indicated in x-axes. siVdH1s (in red vertical lines) above and below the 0 value of y-axes indicate the sense and antisense orientation, respectively. (**b**) Length distribution of siVdH1s in *35S-VdH1i* cotton and Vd^VdH1i−1st^ colony. (**c**) Length distribution of cotton endogenous known miRNAs from *35S-VdH1i* transgenic plants. The repeats are provided in Supplementary data.

The sequencing data revealed significant amounts of siVdH1s of different lengths from 18 to 29 nucleotides (nt) produced in *35S-VdH1i* transgenic cotton. A small amount of siVdH1s was detected in Vd^VdH1i−1st^ colonies but barely in Vd^WT−1st^ colonies (Figure 1a). Similar results were obtained in three individual sequencing libraries of each genotype of the colony (Supplementary Figure S1). The distribution of siVdH1s obtained from *35S-VdH1i* transgenic cotton and Vd^VdH1i−1st^ colonies was aligned with *VdH1* to the region used in designing hairpin RNA for creating *35S-VdH1i* cotton plants [33]. siVdH1s were located alongside both strands of the VdH1i region (Figure 1a,b, and Supplementary Figure S1) but not beyond the VdH1i region, indicating that no transitive siRNAs beyond the RNAi trigger region were generated in recipient hyphae (Figure 1a). Interestingly, the length of siVdH1s was mainly 20 to 23 nt, especially 20 nt, in *35S-VdH1i* transgenic cotton plants and Vd^VdH1i−1st^ colonies, and 24 nt siVdH1s were minimally detected (Figure 1b). This result prompted us to analyze endogenous known miRNAs in plants from sRNA sequencing libraries. As expected, the length of cotton endogenous known miRNAs from *35S-VdH1i* transgenic cotton plants was mainly 21 and 22 nt (Figure 1c), indicating that the preparation and sequencing processes of sRNAs were appropriate. Our data suggest the possible existence of special DCL or relevant protein(s) implemented in processing exogenous RNAi constructs in cotton plants, resulting in the production of 20 to 23 nt siRNAs. Nevertheless, our data confirm that *35S-VdH1i* transgene-derived siRNAs produced in cotton plants were exported to fungal hyphae to induce *VdH1* gene silencing but did not trigger transitive target gene silencing in recipient *V. dahliae*.

### 2.2. Target Gene Silencing Is Reduced over Time in In Vitro Cultured Hyphae Recovered from Infected Plants

**Figure 2:**
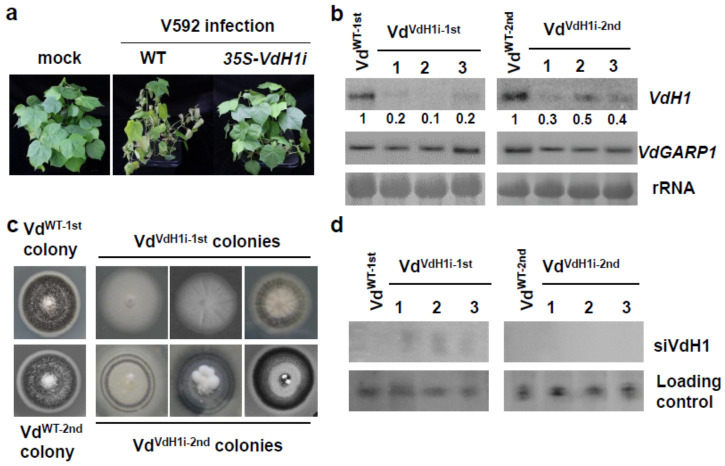
Examination of *VdH1* mRNA and siVdH1 in hyphae recovered from infected cotton. (**a**) Disease symptoms of V592 infection on wild-type and transgenic cotton plants. (**b**) Detection of *VdH1* mRNA in colonies recovered from wild-type and *35S-VdH1i* transgenic cotton plants. The numbers below represent relative signal intensities. Hyphae grown from stems at 5 days post-culture were transferred to PDA medium to continue growth, named Vd^VdH1−1st^ colonies. Subculture colonies propagated from hyphae of Vd^VdH1−1st^ colonies were named Vd^VdH1−2nd^ colonies. (**c**) Morphologies of Vd^VdH1−1st^ colonies and Vd^VdH1−2nd^ colonies. Photographs were taken 20 days post-culture. (**d**) Small RNA hybridization of siVdH1 in Vd^VdH1i−1st^ colonies and Vd^VdH1i−2nd^ colonies.

Given that no transitive silencing occurred in recipient fungal cells, we thus investigated whether target gene silencing in fungal cells would be lastingly maintained or transitorily after recovery from the infected host plants. The sixth generation of transgenic *35S-VdH1i* cotton line 3 was used for V592 infection. Compared to the typical leaf wilt disease symptoms observed for wild-type cotton plants at 20 days postinoculation (dpi) (Figure 2a), transgenic *35S-VdH1i* cotton plants exhibited significantly reduced disease grade in inoculated seedlings (Figure 2a). Consistent with our previous finding [33], *VdH1* mRNA degradation in hyphae recovered from infected *35S-VdH1i* cotton but not wild-type cotton plants was detected (Figure 2b). No significant difference in control Vd*GARP1* mRNA was detected in any of the recovered colonies (Figure 2b). The colony that grew from infected wild-type cotton plants (Vd^WT−1st^) produced normal melanized microsclerotia (Figure 2c). In contrast, colonies that grew from *35S-VdH1i* cotton plants (Vd^VdH1i−1st^) developed *VdH1* knockout mutant-like morphology that lacked or exhibited reduced development of melanized microsclerotia in 20-day-old plate cultures (Figure 2c). 

To examine the maintenance of *VdH1* silencing in recovered hyphae, we selected a patch of the first generational colonies from Vd^WT−1st^ and Vd^VdH1i−1st^ and transferred them to new plates for culture for 20 additional days. Intensive melanized microsclerotium growth was observed for Vd^WT−2nd^. However, Vd^VdH1i−2nd^ colonies exhibited various degrees of melanized microsclerotium growth, and the morphologies differed from those of wild-type V592 (Figure 2c). Weak but clear signals of *VdH1* mRNA were detected in Vd^VdH1i−2nd^ colonies (Figure 2b), which is consistent with the observation of the development of melanized microsclerotia in the Vd^VdH1i−2nd^ colonies. Furthermore, siVdH1s signals were observed in Vd^VdH1i−1st^ colonies but not in Vd^VdH1i−2nd^ colonies (Figure 2d) in accordance with *VdH1* mRNA accumulation in Vd^VdH1i−1st^ and Vd^VdH1i−2nd^ colonies (Figure 2b). Taken together, our data demonstrate that target gene silencing may be maintained transitorily after fungal hyphae depart from host plants. As a result, we speculate that the sustained host-exported dsVdH1/siVdH1s were required and sufficient for silencing *VdH1* in fungal cells inside the host plants, which maintained effective resistance of transgenic *35S-VdH1i* cotton plants. 

### 2.3. Vascular Tissue Is the Most Efficient Location for Trans-Kingdom RNAi between Plants and Vascular Fungi

**Figure 3:**
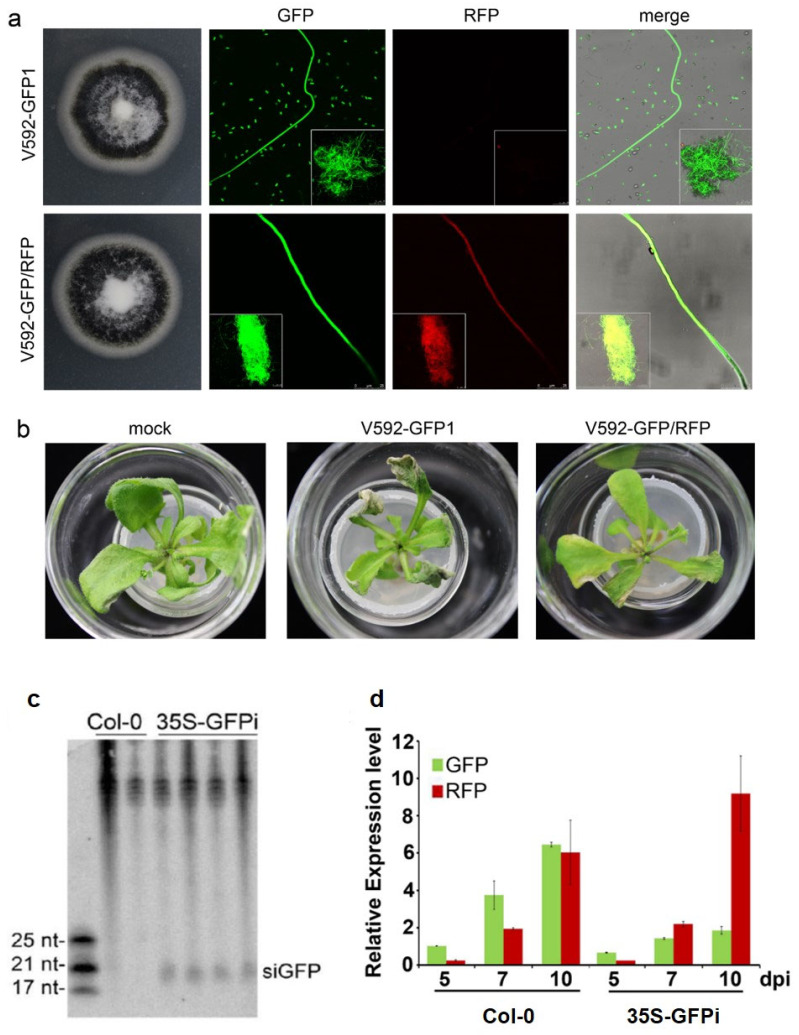
Examination of V592-GFP/RFP in *35S-GFPi* transgenic *Arabidopsis*. (**a**) Colony morphologies and confocal microscopy images of hyphae of V592-GFP1 and V592-GFP/RFP. (**b**) Similar infectivity of V592-GFP1 and V592-GFP/RFP in *Arabidopsis*. Photographs were taken at 10 days postinoculation. (**c**) Detection of GFP-derived siRNAs (siGFP) in *35S-GFPi* transgenic *Arabidopsis*. Total RNAs isolated from wild-type *Col-0* and 4 individual transformants were loaded. The GFP-specific sequence was used as a probe. The upper unspecific bands visible in the top blot serve as the loading control. Positions of 17, 21, and 25 nt siRNAs are indicated. (**d**) Expression of *GFP* and *RFP* mRNAs in infected *Col-0* and *35S-GFPi Arabidopsis*. Total RNAs isolated from roots of ten infected plants at indicated time points were quantified by qRT–PCR and normalized with the corresponding input RNA and *VdELF1* (as the internal standard) levels. The value of *GFP* mRNA in V592-GFP/RFP-infected *Col-0* was arbitrarily designated as 1. Error bars represent SD for three replicates. Reduced levels of *GFP* mRNA but not *RFP* mRNAs were detected in *35S-GFPi*.

**Figure 4:**
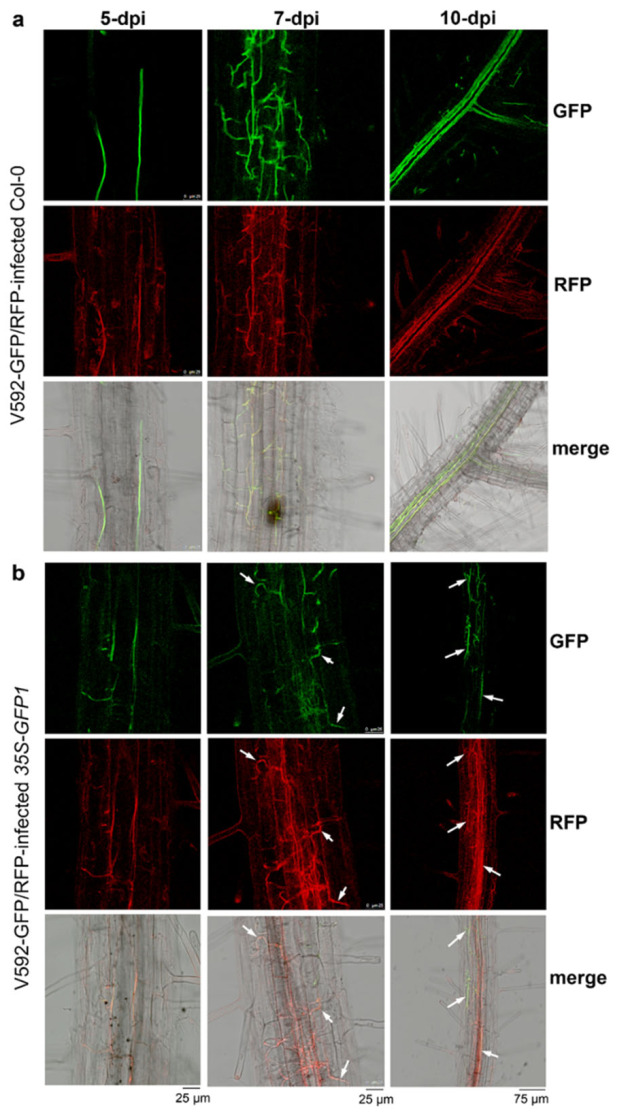
Confocal laser scanning microscopy observation of GFP silencing in *V. dahliae* within infected *Arabidopsis* roots. (**a**,**b**) Confocal laser scanning microscopy images of GFP and RFP signals in hyphae within wild-type (*Col-0*) (**a**) and *35S-GFPi* transgenic (**b**) Arabidopsis roots infected by double-labeled *V. dahliae* isolate V592-GFP/RFP. The merged images are compound micrographs of bright field transmission and the corresponding GFP and RFP fluorescence images. Examples of hyphae overlapped with both GFP and RFP signals are indicated by arrows. Six images for each root sample were observed and similar results were obtained.

To test our speculation on the efficient trans-kingdom silencing in fungal cells inside the infected plants, we first created a visible HIGS system. GFP-labeled V592 (V592-GFP1) [46] was retransformed with *pSulPH-RFP*, producing V592-GFP/RFP with similar growth morphology to V592-GFP1 (Figure 3a). Similar disease severity in *Arabidopsis* was caused by V592-GFP1 and V592-GFP/RFP. Leaf wilt was observed at 10 dpi (Figure 3b), revealing the normal infectivity of V592-GFP/RFP. To achieve visible HIGS of *GFP* in the hyphae, *35S-GFPi* transgenic *Arabidopsis* was created. The production of GFP-derived siRNAs (siGFP) in *35S-GFPi* transgenic *Arabidopsis* was confirmed (Figure 3c). Wild-type and *35S-GFPi* transgenic *Arabidopsis* were inoculated with spores from V592-GFP/RFP by unimpaired root-dip inoculation [46]. Confocal laser scanning microscopy (CLSM) revealed that both GFP and RFP signals were observed in hyphae within the root cortical tissue in wild-type *Arabidopsis* by 5 dpi (Figure 4a). Both green and red fluorescence were also observed within the root cortical tissue in *35S-GFPi* plants by 5 dpi; however, the GFP signal was obviously reduced, resulting in stronger red fluorescence in the merged images than that noted in wild-type *Arabidopsis* (Figure 4b). Similarly, while stronger GFP signals relative to RFP signals were observed in the hyphal net within the vascular tissue in wild-type *Arabidopsis* by 7 dpi (Figure 4a), stronger RFP relative to GFP signals in the hyphal net within the vascular tissue were observed in *35S-GFPi* plants by 7 dpi (Figure 4b). Two signals overlapping in some hyphae were still observed in *35S-GFPi* plants (Figure 4b, arrows). Again, intense GFP signals were observed in rapidly proliferating hyphae within the xylem vessel in wild-type *Arabidopsis* by 10 dpi (Figure 4a). In contrast, intense RFP signals were observed in most hyphae within the xylem vessel in *35S-GFPi* plants (Figure 4b); only a few hyphae outside or around the vascular bundle maintained a clear GFP signal and overlapped with the RFP signal, as shown in the merged image (Figure 4b, arrows). Consistently, reduced levels of *GFP* mRNA but not *RFP* mRNA were detected by qRT–PCR of total RNAs extracted from the roots of the ten inoculated *35S-GFPi* plants at various time points (Figure 3d). Taken together, our results clearly demonstrate that hyphae in *35S-GFPi* plants were effectively and specifically triggered for *GFP* silencing, and *GFP* silencing was effectively sustained in the rapid proliferation of hyphae within the vascular tissue but possibly less effective within the cortical tissue of *Arabidopsis* roots where hyphae intercellularly crossed to reach the vascular tissue to realize colonization [46].

## 3. Discussion

The control strategy of fungal pathogens relies on the use of fungicides that are harmful to the environment. The newly developed HIGS is based on a sequence-specific RNAi mechanism that is friendly to the environment. Although considerable progress has been achieved in utilizing the HIGS strategy to protect against plant fungal pathogens, further research evaluating the efficiency, stability, and durability of resistance is needed in the field, thereby improving our understanding and application of this new approach.

In this study, small RNA sequencing results showed that siVdH1s from hyphae recovered from *V. dahliae*-infected *35S-VdH1i* cotton matched but not beyond the VdH1i region (Figure 1a). Additionally, we showed that trans-kingdom VdH1i-derived siVdH1s-mediated degradation of *VdH1* exists in hyphae fresh recovered from infected *35S-VdH1i* cotton plants, given that the colonies of the Vd^VdH1i−2nd^ passages from recovered Vd^VdH1i−1st^ resumed normal *VdH1* accumulation (Figure 2b). These data suggest the lack of transitive silencing in *V. dahliae* and indicate that three fungal RDR proteins are not involved in the trans-kingdom siRNA-induced silencing in *V. dahlia* [7]. This is partly similar to that in *Rosellinia necatrix*, a plant pathogenic fungus, in which systemic RNAi is not triggered by locally induced RNAi [47]. It has also been reported in *F. asiaticum* that siRNAs were only matched to the exogenous dsRNA triggers but not the target mRNA beyond the dsRNA trigger regions, indicating that RDR-dependent secondary siRNA amplification does not occur. Moreover, exogenous dsRNA exhibited a similar silencing efficiency in RDR mutants compared with that in wild-type [48]. These findings provide clues to explain the lack of secondary siRNA amplification as a divergent function of RDRs in fungi. Although there seems to be no RNAi amplification in *V. dahliae*, the siVdH1s generated persistently in transgenic *35S-VdH1i* cotton are sufficient to silence the *VdH1* gene to impart durable resistance. This finding differs from that noted for exogenously applied dsRNA or siRNAs, for which RNA amounts represent a limiting factor. 

In plants, RNAi signals are transmitted locally from cell to cell through plasmodesmata (PD) and over long distances through the phloem [49,50]. The plant phloem may represent a site of accumulation of mobile sRNAs, the levels of which are modulated by stress conditions [51]. It is worth noting that the vascular fungal pathogen *V. dahliae* can efficiently take up host-derived sRNAs during infection [39], which may be due to the location of pathogen colonization. Hence, the plant vasculature might represent a target site for effective trans-kingdom RNAi. Notably, in V592-GFP/RFP-infected *35S-GFPi Arabidopsis* roots, we observed strong GFP silencing of V592-GFP/RFP in vascular tissues, where strong red fluorescent hyphae were noted, but a lesser silencing effect in cortical tissues, where green/red fluorescent hyphae were present (Figure 4b). This finding is consistent with the role of mobile siRNAs in long-distance silencing through the plant vascular system. This result again suggests that silencing in hyphae is attributed to host-delivered siRNAs in fungal cells. Remarkably, whereas strong green hyphal proliferation was noted in wild-type *Arabidopsis* roots (Figure 4a), red hyphae were observed in the vascular tissue of *35S-GFPi Arabidopsis* roots (Figure 4b). These results indicate that once GFP silencing in hyphae was established, it was maintained during hyphal growth and proliferation in vascular tissue. The formidable proliferation of *V. dahliae* hyphae in plant vascular tissue [46] together with the driving force of phloem flow underlying the long-distance transport of the silencing signal endows HIGS with high efficiency in cotton protection against the vascular pathogen *V. dahliae*.

In recent years, SIGS, or exogenously applied dsRNA or siRNAs in host plant protection, has become popular. A few studies revealed that SIGS for disease control was dependent on the efficiency of pathogen RNA uptake [43] and different dsRNA application approaches [52]. Additionally, an increasing number of studies have focused on improving strategies for prolonged dsRNA stability, efficacy, and scalability, such as *Escherichia coli*-derived anucleated minicells for dsRNA production and encapsulation [53], laser-assisted delivery of dsRNA [54], and nanoparticles for potential delivery of siRNA [54]. Although these GMO-free RNAi strategies are convenient and unlabored, the application frequency and amounts are factors that need to be considered and evaluated. Transgene-based HIGS possesses a distinctive advantage, such as constant delivery of siRNAs from plants to pathogens, ensuring the siRNA supply needed to trigger trans-kingdom RNAi. Nevertheless, exogenous application or constitutive expression of dsRNA or siRNAs is worthy of development for crop protection. Our study demonstrates that HIGS effectively triggers long-lasting trans-kingdom RNAi during interactions between transgenic cotton plants and *V. dahliae*, despite no amplification of RNAi being noted in this soil-borne fungal pathogen. Exploring the fungal endogenous RNAi pathways is needed to further reveal the molecular basis for this trans-kingdom RNAi, thus helping for better utilization of HIGS in crops. On the other hand, more pathogenic genes need to be tested for ideal targets in trans-kingdom RNAi. In all, our work provides further understanding of the efficacy of HIGS in defending against plant soil-borne vascular pathogens. In the future, fundamental knowledge on the molecular mechanisms of HIGS and SIGS will lead to novel integrative approaches or tailor-made solutions for controlling plant diseases [55]. 

## 4. Materials and Methods

### 4.1. Fungal Isolates, Culture Conditions, and Fungal Recovery and Infection Assays

A virulent defoliating *V. dahliae* isolate V592 from cotton was used in this study. The culture conditions of V592, the conidia production for infection assays, and the fungal recovery assay in cotton were described previously [33,56]. For plant infection assays in the laboratory, the “laboratory unimpaired root-dip inoculation method” described previously [56] was used for cotton and *Arabidopsis* root inoculation.

### 4.2. Cloning and Constructs

For the *35S*-*GFPi* RNAi constructs, the sense and antisense sequences of the 3′-terminal 500 bp of GFP were amplified by PCR using sequence-specific primers as follows: forward primer, 5′-GGATCCATGCCGTGAGTGATCCCG-3′ (underlined letters: BamHI site); reverse primer, 5′-GAATTCGTGCTTCAGCCGCTACCC-3′ (underlined letters: EcoRI site). The antisense sequence was amplified using forward primer, 5′-GAGCTCATGCCGTGAGTGATCCCG-3′ (underlined letters: SacI site); reverse primer, 5′-AGATCTGTGCTTCAGCCGCTACCC-3′ (underlined letters: BglII site). Each of the PCR fragments was ligated into the pGEM-T vector (Tiangen). Sense and antisense sequences were inserted into an intron-containing intermediate construct (pSK-int) [57] to obtain sequence cassettes containing the inverted-repeat RNAi constructs as previously described [57], producing pSK-GFPi. A fragment of BamHI-SacI from pSK-GFPi was inserted into the binary vector pBI121 under the 35S promoter to generate *35S*-*GFPi* for plant transformation. 

To create double-labeled GFP and RFP of the *V. dahliae* isolate, pSulPH-RFP-NEO, which contains a neomycin (neo) resistance cassette, was generated as follows: the neo resistance cassette was amplified from pKOV21 [58] with primers 5′-GCTCTAGACAGCCGCCTTCGCAAGCGCT-3′ and 5′-GCTCTAGAGGCCAGCAGTAGACACTTGG-3′ (underlined letters: XbaI site). The PCR fragment was ligated into the pGEM-T easy vector, and the XbaI-digested fragment was inserted into XbaI-digested pSulPH, conferring the resulting pSulPH-NEO resistance to geneticin. The RFP fragment was amplified from the pGDR vector [59] with primers 5′-GCGGATCCATGGCCTCCTCCGAGAACGT-3′ (underlined letters: BamHI site) and 5′-GAATTCGCGATGTCCTTGTCCACCACCG-3′ (underlined letters: EcoRI site). The PCR fragment was inserted into pSulPH-NEO, resulting in pSulPH-RFP-NEO, which was retransformed to V592-GFP1 [46], producing the double-labeled *V. dahliae* isolate V592-GFP/RFP.

### 4.3. Fungal and Plant Transformation

The *35S*-*GFPi* constructs were transformed into the *Agrobacterium* strain *EHA105* for plant transformation. *Arabidopsis* (Columbia ecotype) was transformed according to the standard floral dip method [60]. The fungal transformation was performed as previously described [61].

### 4.4. RNA Extraction, RNA Gel Blotting, and Quantitative Real-Time PCR Analysis

Fungal isolates were grown in liquid Czapek–Dox medium for 3 days with shaking at 200 rpm and 26 °C in the dark, and the resulting mycelium was harvested for RNA isolation using TRIzol reagent (Invitrogen) according to the manufacturer’s instructions. For high molecular weight RNA gel blots, 20 µg of total RNA was separated on 1.2% agarose gels containing 6% formaldehyde and transferred to nylon N+ membranes. DNA probes were labeled with [a-^32^P] dCTP using the Rediprime II system (Amersham). For low molecular weight RNA gel blots, 40 µg of total RNA was separated by electrophoresis on 17% PAGE gels and electrically transferred to nylon N+ membranes. Then, [ɑ-^32^P] UTP-labeled gene-specific transcript sequences were used (New England Biolabs). For detection of the silencing of *VdH1* in recovered hyphae, colonies recovered from the same infected plant at 20 days postinoculation were mixed for RNA isolation. For qRT–PCR, 2 μg of total RNA was reverse transcribed into cDNA using HiScript II Q RT SuperMix for qPCR (Vazyme). qRT–PCR analysis was performed with a 1000 series Thermal Cycling Platform (Bio-Rad) using SYBR qPCR Master Mix (Vazyme). The constitutively expressed elongation factor 1-α of *V. dahliae* (*VdELF1*) was used as an internal control. Gene-specific primers are listed below: VdELF1-qRT-F, CCATTGATATCGCACTGTGG and VdELF-qRT-R, TGGAGATACCAGCCTCGAAC; RFP-qRT-F, AGGACGGCTGCTTCATCTAC and RFP-qRT-R, CTTCAGGGCCTTGTGGGT; GFP-qRT-F, ATGGTGAGCAAGGGCGAGGAG and GFP-qRT-R, TAGGTCAGGGTGGTCACGAGG. At least three biological replicates and three technical replicates were performed in each experiment for each sample. 

### 4.5. Confocal Laser Scanning Microscopy

A. thaliana roots were immersed in a conidial suspension (~10^5^ conidia/mL in water solution) for 10 min and then transferred onto a 0.75% agar plate at 25 °C in the dark. Images were obtained under a confocal laser microscope (Leica TCS SP8; Leica Microsystems) with 100× oil immersion objective lenses. The excitation wavelengths and emission filters were as follows: 488 nm/bandpass 500 to 550 nm for GFP and 561 nm/bandpass 570 to 670 nm for RFP. Confocal images were captured with a Leica hybrid detector and analyzed with Leica LAS AF software. 

### 4.6. Small RNA Sequencing

*V. dahliae* recovered from V592-infected cotton (Vd^WT−1st^) and V592-infected *35S-VdH1i* cotton (Vd^VdH1i−1st^) were grown in liquid Czapek–Dox medium and harvested as described above for RNA extraction. *35S-VdH1i* transgenic cotton plants grown at 26 °C with a 16 h light (8000 lux)/8 h dark cycle for approximately 3 weeks were subject to RNA extraction. RNA isolation, sRNA library construction, and sRNA sequencing were performed by BGI (http://www.bgitechsolutions.com/, accessed on 11 February 2022). The raw data were filtered to remove low-quality reads to obtain clean sequences. The sRNAs were aligned with *VdH1* to the region used in designing hairpin RNA for creating *35S-VdH1i* cotton plants. A Perl script was used to search for known miRNAs in cotton [62,63] with 18–30 nt clean sequences. The expression level of miRNAs was normalized by RPM.

## Data Availability

Not applicable.

## Supplementary Materials

The following supporting information can be downloaded at: https://www.mdpi.com/article/10.3390/ijms23052742/s1.
