# Supplementary material for: Exploring the Effectiveness and Durability of Trans-Kingdom Silencing of Fungal Genes in the Vascular Pathogen Verticillium dahliae
Source: Int J Mol Sci. 2022 Mar 1;23(5):2742. doi: 10.3390/ijms23052742 (PMC8910871; doi:10.3390/ijms23052742)
Supplement: Supplementary file 1 [file ijms-23-02742-s001.zip › Figure S1 legend.pdf]

Figure.S1. The two repeats of sRNA sequencing analysis data shown in Figure.1 (a)-(c) for one repeat, and (d)-(f) for another repeat. (a) and (d) RPM and distribution of siVdH1s obtained by deep sequencing. (b) and (e) Length distribution of siVdH1s in *35S-VdH1i* cotton and $Vd^{VdH1i-1st}$ colony. (c) and (f) Length distribution of cotton endogenous known miRNAs from *35S-VdH1i* transgenic plants.
