# Supplementary figures and images for: Exploring the Effectiveness and Durability of Trans-Kingdom Silencing of Fungal Genes in the Vascular Pathogen Verticillium dahliae
Source: Int J Mol Sci. 2022 Mar 1;23(5):2742. doi: 10.3390/ijms23052742 (PMC8910871; doi:10.3390/ijms23052742)

# **VdH1 mRNA (584bp)**

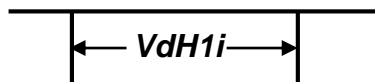

**a**

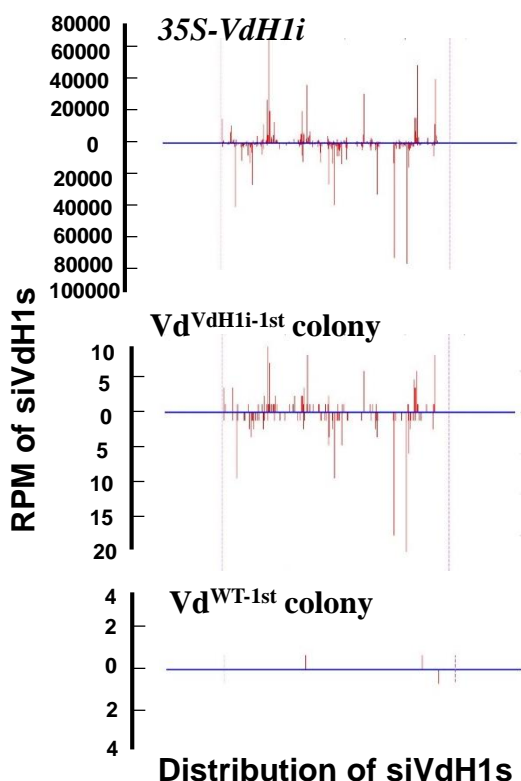

**b**

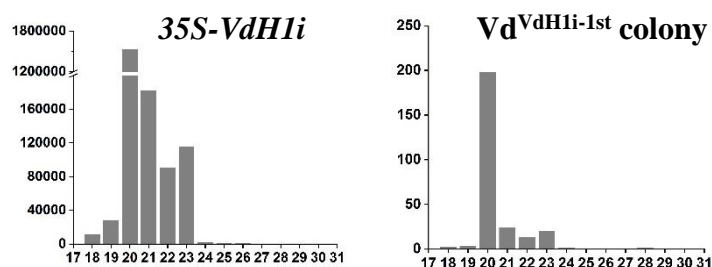

**c**

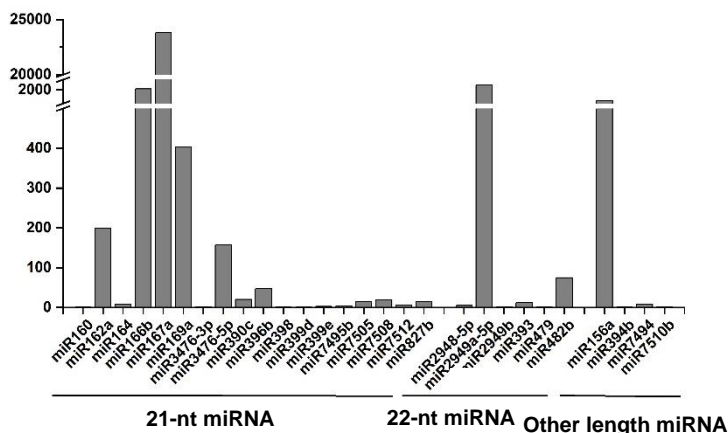

**d**

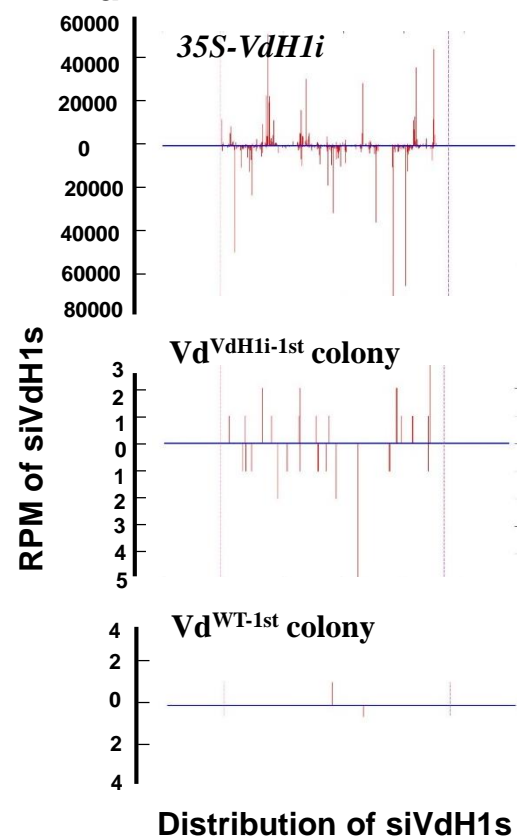

**e**

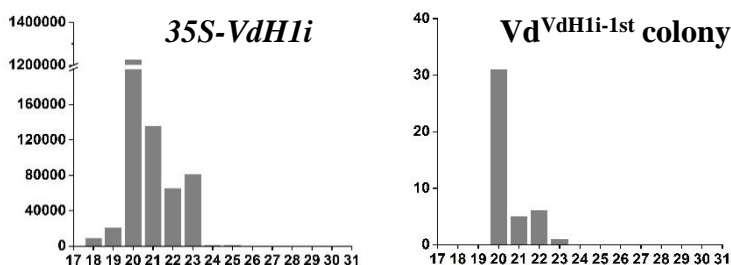

**f**

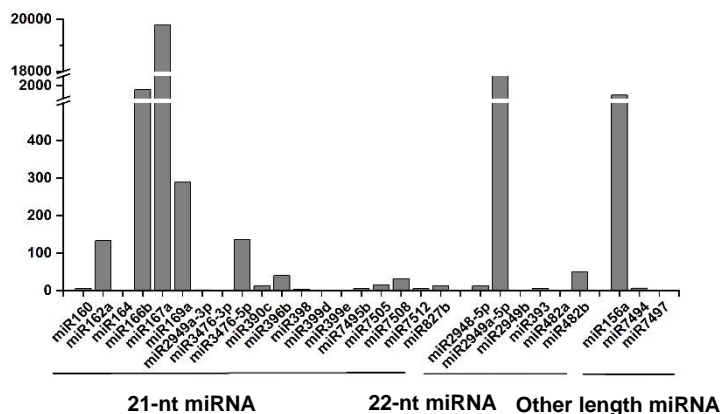

Supplement: Supplementary file 1 [file ijms-23-02742-s001.zip › Figure S1.pdf]
